# Single Crystal Perovskite/Graphene Self-Driven Photodetector with Fast Response Speed

**DOI:** 10.3390/ma17112599

**Published:** 2024-05-28

**Authors:** Xiao Liu, Xiangshun Geng, Guanhua Dun, Zeshu Wang, Jingbo Du, Dan Xie, Yi Yang, Tianling Ren

**Affiliations:** The Beijing National Research Center for Information Science and Technology (BNRist), School of Integrated Circuits, Tsinghua University, Beijing 100084, China

**Keywords:** perovskite, graphene, heterostructure, self-driven photodetector

## Abstract

Recently, the combination of two-dimensional (2D) materials and perovskites has gained increasing attention in optoelectronic applications owing to their excellent optical and electrical characteristics. Here, we report a self-driven photodetector consisting of a monolayer graphene sheet and a centimeter-sized CH_3_NH_3_PbBr_3_ single crystal, which was prepared using an optimized wet transfer method. The photodetector exhibits a short response time of 2/30 μs by virtue of its high-quality interface, which greatly enhances electron–hole pair separation in the heterostructure under illumination. In addition, a responsivity of ~0.9 mA/W and a detectivity over 10^10^ Jones are attained at zero bias. This work inspires new methods for preparing large-scale high-quality perovskite/2D material heterostructures, and provides a new direction for the future enhancement of perovskite optoelectronics.

## 1. Introduction

Photodetectors are of central importance to various applications, such as military surveillance, optical communications, automobiles, environmental detection, and biomedical sensing. In recent years, perovskite/two-dimensional (2D) material heterojunction photodetectors have become a popular research topic [1,2,3,4]. Perovskite materials have drawn increasing attention over the past decade owing to their excellent optical and electronic properties, such as their high optical absorption coefficient, long carrier lifetime and diffusion lengths, tunable band gap, and low-cost processing. Meanwhile, since the discovery of graphene, the potential of 2D layered materials in optical detection has attracted intense interest due to their distinctive mechanical, optical, and electrical characteristics. In a perovskite/2D material heterojunction photodetector, the perovskites act as the active layer, absorbing photons to generate electron–hole pairs. The photoexcited carriers are efficiently separated at the interface of the heterostructure by the built-in electric field [5,6]. Benefiting from the high absorption coefficient of perovskites and the high carrier mobility of graphene, these heterojunction photodetectors could achieve a high photoresponsivity, a fast photoresponse, and a large dynamic range [7,8]. The combination of these two kinds of materials promises better performance than 2D-material-only photodetectors, as the atomic-scale thickness of 2D materials significantly restricts their light absorption capacity. The photoresponsivity of perovskite/graphene could exceed hundreds of A/W [9], which is better than that of pure graphene photodetectors [10] by several orders of magnitudes. Furthermore, compared with perovskite-only photodetectors, the combination of these two materials in the device prevents the direct exposure of perovskites to the environment, leading to better resistance to moisture and oxygen. Therefore, the perovskite/2D material heterostructure is a good candidate for high-performance photodetectors.

Graphene, as a typical 2D material, is promising for building high-performance perovskite/2D-material heterostructures. Clean, residue-free, and highly conductive graphene is highly desirable for promoting interfacial coupling and charge injection for heterojunction photodetectors. The conventional method of preparing graphene on a desired substrate often requires a growing process (generally, chemical vapor deposition (CVD) on transition metal substrates) and a subsequent transfer process [11,12]. During the transfer process, the growth substrate is removed by dissolution in a liquid etchant, bubble delamination, or thermal peel-off [13], and then the graphene is transferred to the target substrate by wet or dry transfer method. A supporting layer composed of organic polymers is often used to prevent mechanical damage. Wet transfer, as an effective means of constructing heterogeneous structures, has the advantages of simple operation, high efficiency, and suitability for large-scale transfer. The choice of the supporting layer and the liquid solvent during the wet transfer process has a large influence on the electrical properties, structural integrity, and uniformity of the graphene. However, the typical liquid transfer solvent (water) and supporting material (polymethyl methacrylate, PMMA) are not compatible with perovskite. Water would induce the degradation of halide perovskites by processes including hydration, phase transformation, decomposition, and dissolution [14,15]. In addition, PMMA is solvable in *N*,*N*-dimethylformamide (DMF), which is one of the most commonly used solutes for the preparation of perovskites. Consequently, a substitute for the supporting layer or modification of the perovskite preparation method must be sought to retain the benefits of the wet transfer method for producing high-quality heterostructures. Using polydimethylsiloxane (PDMS) and saturated perovskite solution as supporting layer and transfer solvent can overcome the drawbacks of the conventional wet transfer method when applied to perovskites. By replacing deionized water with a saturated perovskite solution, the degradation of perovskites can be avoided. Similar to PMMA, PDMS is a kind of organic polymer material exhibiting good chemical stability, appropriate mechanical strength, and high transparency, and can be easily prepared using the spin-coating technique. More importantly, PDMS is not solvable in DMF and so can be compatibly integrated into perovskite fabrication processes. Through these optimizations, the wet transfer technique could be applied to perovskites, and heterostructures with a large size and high-quality interfaces can be prepared.

Here, we demonstrate a self-driven perovskite/graphene heterostructure photodetector exhibiting fast responses via an optimized wet transfer method, which can transfer large-size graphene onto perovskite single crystals. Polydimethylsiloxane (PDMS) and saturated perovskite solution were used as the supporting layer and transfer solvent. MAPbBr_3_ is used as the light-harvesting material, considering its good crystallinity and excellent photoelectric conversion capability for visible light. On the heterojunction interface prepared by the optimized method, the photodetector exhibits a responsivity of ~0.9 mA/W, a detectivity over 10^10^ Jones at zero bias, and a fast response speed of 2/30 μs. This work sheds new light on perovskite/2D material heterostructures in optoelectronic applications.

## 2. Materials and Methods

### 2.1. Materials

Lead bromide (PbBr_2_) and *N*,*N*-dimethylformamide (DMF) were purchased from Aladdin (Shanghai, China). Methylammonium bromide (CH_3_NH_3_Br) was purchased from Greatcell Solar (Queanbeyan, Australia). Dichloromethane (DCM) was purchased from Energy Chemical (Shanghai, China). The monolayer graphene film on copper foil synthesized by CVD was purchased from XFNano (Nanjing, China). All the materials were used directly without further purification.

### 2.2. Synthesis of Perovskite Single Crystal

**Figure 1:**
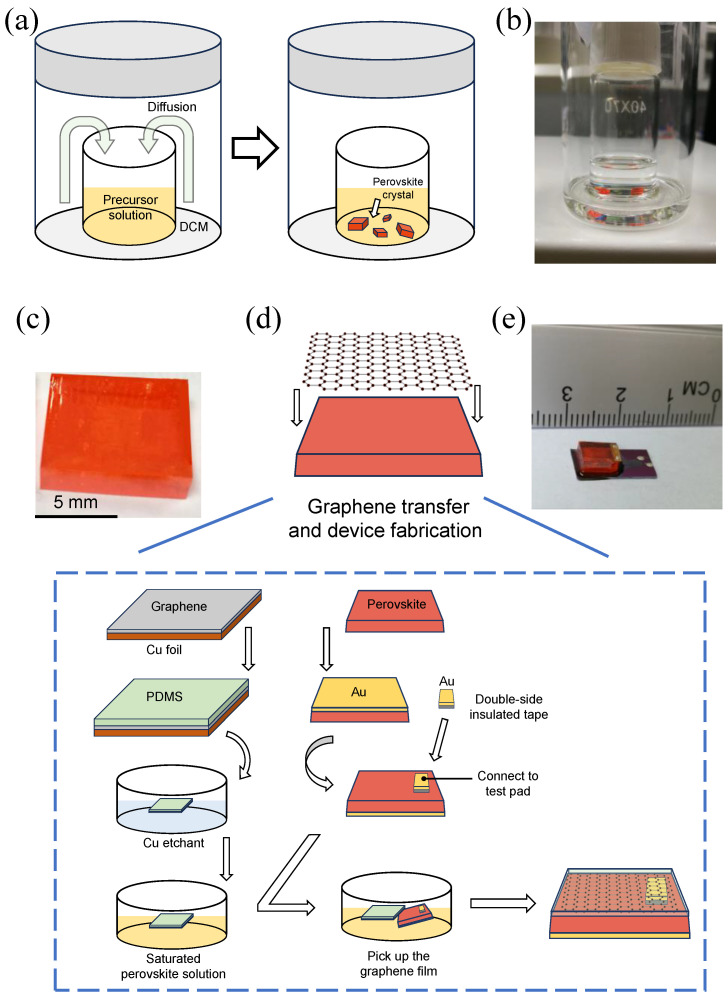
Schematic diagram of device fabrication: (**a**) Growth of perovskite by antisolvent crystallization method. (**b**) Optical photograph of the growth apparatus. (**c**) Optical photograph of the perovskite crystal. (**d**) Schematic diagram of the device fabrication. (**e**) Optical photograph of the photodetector.

The CH_3_NH_3_PbBr_3_ (MAPbBr_3_) perovskite single crystal was synthesized by a reported antisolvent crystallization process [16,17]. The precursor solution was prepared by dissolving PbBr_2_ and MABr in DMF in a 1:1 molar ratio and stirring for 1 h. The vial containing precursor solution was partially sealed, leaving a small hole to allow DCM to slowly diffuse in, and then placed in a larger outer vial containing DCM (Figure 1a). DCM was used as an antisolvent to precipitate the single crystals. The outer vial was fully sealed and stored in darkness. Thereafter, the MAPbBr_3_ single crystals grew slowly for several days as the DCM diffusing into the inner vial (Figure 1b). The crystal shown in Figure 1c has a regular square shape in semitransparent red with a size of 9 mm × 9 mm × 1.4 mm.

### 2.3. Transfer of Graphene and Device Fabrication

The heterojunction photodetector was fabricated in the device stack Au/MAPbBr_3_/graphene/Au. The processing steps of wet transfer process and device fabrication are illustrated in Figure 1d. A gold-bottom electrode was deposited on the bottom surface of the MAPbBr_3_ single crystal with a shadow mask by sputtering. At the same time, a gold layer was deposited on a double-sided tape, which was then stuck to the top surface of the perovskite single crystal as the top electrode. The crystal with top and bottom electrodes was latterly used as the target substrate in the following wet transfer process.

The high-quality graphene was transferred to the target substrate through a polymer-assisted wet transfer process. The polydimethylsiloxane (PDMS) layer was spin-coated onto the graphene/copper surface (3000 r/min for 30 s) and baked at 120 °C for 5 min. Then, the film was placed into the ammonium persulfate etchant to etch the copper foil. The floating graphene supported by the PDMS layer was picked up with a clean glass slide and washed in deionized water 3 times. After that, the graphene/PDMS film was transferred again into DMF and washed several times to eliminate the residual water. The film was then transferred to saturated perovskite precursor solution. Finally, MAPbBr_3_ single crystal was used as the target substrate to pick up the floating graphene film in the saturated perovskite solution. The PDMS supporting layer was not necessary to remove in our device structure. In addition, it can function as an encapsulating layer to prevent dust and mechanical damage. Figure 1e shows an optical photograph of the prepared photodetector; the size of the perovskite crystal used in this device is about 6 mm × 6 mm × 2 mm.

Another photodetector based on pure MAPbBr_3_ single crystal was also fabricated for comparison. Gold electrodes were deposited on the side surfaces of the perovskite with the shadow mask by sputtering. The structure diagram and optical photograph of this device are shown in Figure S1 (Supplementary Material).

### 2.4. Characterization Methods

The topography and morphological features of the photodetector were investigated by optical microscope and scanning electron microscope (SEM, GeminiSEM 300, Zeiss, Oberkochen, Germany). The X-ray diffraction spectra were collected with a scan step of 0.02° using a D/max-2550 X-ray diffractometer (Rigaku, Akishima, Japan) and Cu Kα irradiation (λ = 1.54056 Å). The UV–Vis absorption spectra were recorded using a U-3010 spectrophotometer (Hitachi, Tokyo, Japan). The photoluminescent (PL) measurements were performed employing a FLS-980 (Edinburgh, Livingston, England) apparatus with an excitation wavelength of 532 nm. The Raman spectra were recorded by a Horiba Jobin Yvon spectrometer equipped with a 532 nm laser. All these characterization methods were performed in general fashion with no additional change in methodology.

All the electrical parameters of the devices were measured with a semiconductor characterization system (Keithley B1500A, Tektronix, Shanghai, China) assisted by a probe station. The monochromatic light was obtained from a semiconductor laser at the wavelength of 520 nm, and a light power meter was used to measure the light intensity.

## 3. Results

**Figure 2:**
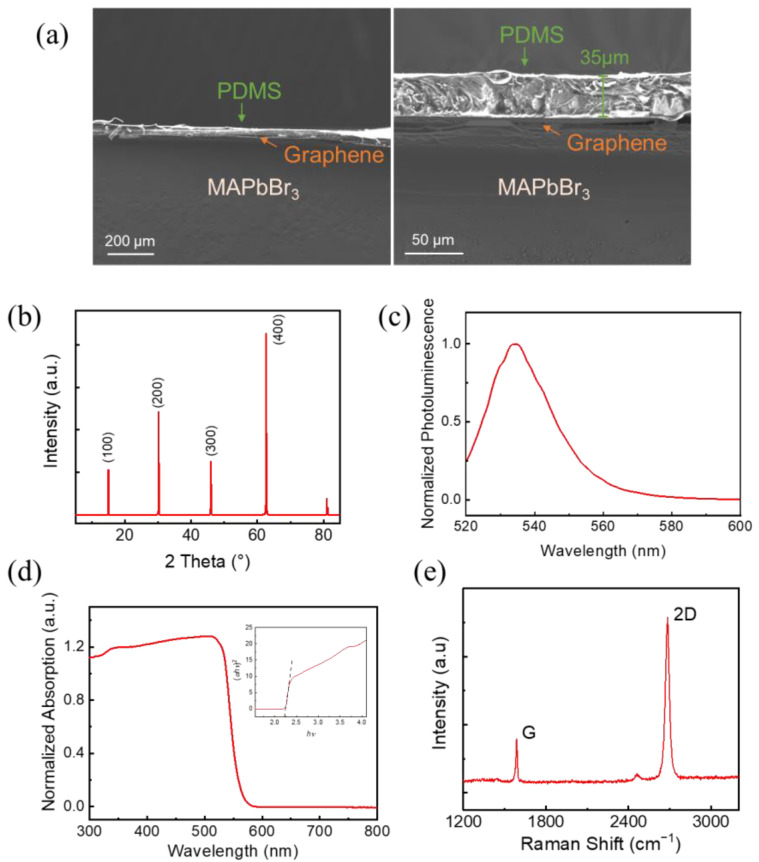
Characterization of the materials: (**a**) The cross-sectional SEM image of the heterostructure. (**b**) The XRD spectrum of perovskite single crystal. (**c**) The PL spectrum of perovskite. (**d**) The UV–Vis absorption spectrum of perovskite and the Tauc plot. (**e**) The Raman spectrum of graphene.

Figure 2a presents the cross-sectional scanning electron microscope (SEM) image of the device at different magnifications. It can be seen that the perovskite crystal demonstrates uniform and smooth morphology at micrometer scale. The perovskite crystal presents good compactness, and no evident voids or cracks can be observed. The PDMS layer is well attached to the surface of perovskite crystal, indicating good contact of the perovskite/graphene interface. The thickness of PDMS measures about 35 μm. Therefore, the influence of light absorption in PDMS is limited, since it holds a high transmittance over 90% in the visible light domain [18]. Figure 2b presents the XRD spectrum of the perovskite crystal; the spectrum shows strong diffraction peaks at 14.96°, 30.14°, 45.92°, and 62.64°, which can be assigned to the (100), (200), (300), and (400) planes of the cubic perovskite structure ($Pm\bar{3}m$), respectively. All the characteristic peaks are in good agreement with the values reported in previous studies [19,20]. No impurity peaks were observed, indicating that no additional phases were formed, and no MABr or PbBr_2_ materials were overstayed. According to these SEM and XRD measurements, the as-grown perovskite crystal has good crystallinity and a monocrystalline structure.

The optical properties of the perovskite were further examined by measuring the steady-state photoluminescence (PL) and UV–Vis absorption spectrum. The perovskite samples used for these optical measurements were grown in the same batch as the one used for the photodetector. The PL spectrum shown in Figure 2c exhibits a peak centered at 534 nm (2.32 eV) with a full width at half maximum (FWHM) of 19 nm, consistent with typical value of MAPbBr_3_ single crystal reported before [21,22]. The optical cutoff wavelength was measured at 565 nm from the absorption spectrum (Figure 2d). The absorption curve has a single straight decline region, and no significant defect level absorption can be observed except the normal Urbach tail, indicating that the perovskite is defect-free. These characteristics are consistent with the direct bandgap nature of MAPbBr_3_ and indicate good quality of the crystal. The value of bandgap is 2.22 eV, obtained by the conventional Tauc plot [23]. That is, we plotted the (*αhν*)^1/2^—*hν* curve and measured the optical bandgap from the intercept of extension line. Since the PL spectrum and UV–Vis absorption spectrum is strongly related to the band-to-band transition of the semiconductor, these results indicate a detection wavelength limit at about 530~560 nm of the perovskites.

Raman measurements were performed using a 532 nm excitation laser source in air. As shown in Figure 2e, the G band and the 2D band of graphene appear at 1587 cm^−1^ and 2684 cm^−1^, respectively. The emergence of the G peak is due to the doubly degenerate zone center mode, and the 2D peak is due to the second order of zone-boundary phonons [24]. The ratio of integrated intensity of the 2D band to the G band *I*_2D_/*I*_G_ is counted to be 5.3, and both bands exhibit a single Lorentzian feature with no splitting, indicating that the graphene is monolayer and holds a high quality [25].

**Figure 3:**
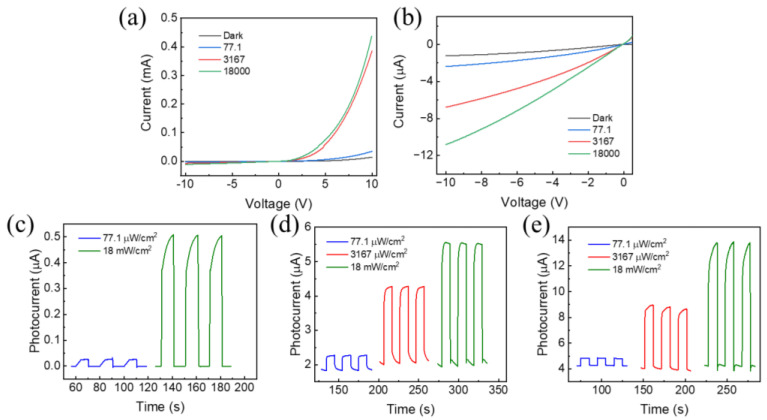
(**a**) *I*–*V* curve of the photodetector. (**b**) *I*–*V* curve of the photodetector under negative bias. (**c**–**e**) Photocurrent of the device under ON–OFF illumination at (**c**) zero bias, (**d**) −4 V bias, and (**e**) −10 V bias.

We constructed a photodetector based on the perovskite/graphene heterostructure, and studied its photoelectric properties. Figure 3a,b illustrate the current–voltage (*I*–*V*) characteristics of the photodetector under light with different incident illumination powers of 520 nm wavelength. The device shows a rectification behavior. Under negative bias, both dark current and photocurrent show mainly linear principle to the bias voltage. Under positive bias, the current grows exponentially with the bias voltage. Low dark currents of −1.21 μA and 14.6 μA were acquired at the voltages of −10 V and +10 V, respectively, and a rectification factor of ~12 can be calculated. The device presents conspicuous light response, as the photocurrent increases with the illumination power. Under the illumination with a light density of 77.1 μW/cm^2^, the photocurrents at the voltage of −10 V and +10 V were recorded to be 2.37 μA and 34.9 μA, respectively, resulting in a rectification factor of ~15. Under the illumination with a light density of 18 mW/cm^2^, the photocurrents at the voltage of −10 V and +10 V were recorded to be 10.8 μA and 438 μA, respectively, resulting in a rectification factor of ~40. The rectification factor becomes larger as the incident light raises. Figure 3c–e are the temporal responses of the photocurrent under switching illumination at fixed applied voltage of 0 V, −4 V, and −10 V. The device exhibits good and stable ON–OFF switching behavior over multiple cycles. It is to be observed that a photocurrent of microampere level exists at zero bias. Therefore, the photodetector can operate without an external driving voltage. In other words, this self-driven photodetector can detect illumination signal without additional energy consumption. The maximum photocurrent at zero bias in our test was 0.5 μA, obtained when the light intensity was 18 mW/cm^2^, and the maximum photocurrent at −10 V was 14 μA under illumination of the same power.

**Figure 4:**
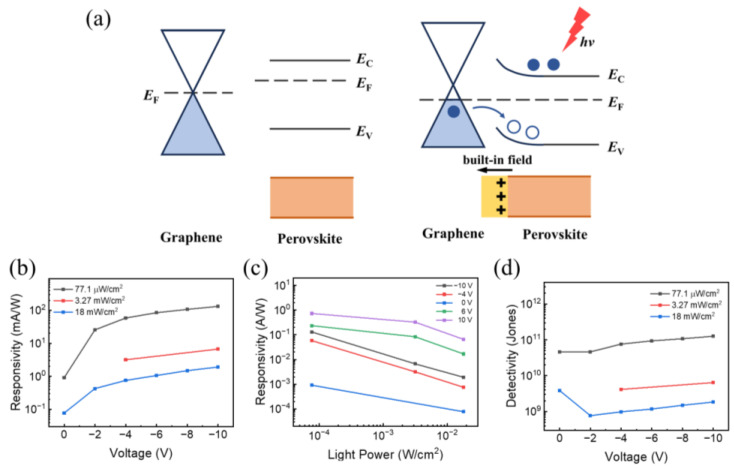
(**a**) Energy band diagram of the perovskite/graphene heterostructure. (**b**,**c**) Photoresponsivity of the device under different light intensity and voltage bias. (**d**) Detectivity of the device under different light intensity.

Figure 4a illustrates the band alignment of the heterostructure, which could explain the self-driven detection capability of the photodetector. The work function of graphene is about 4.6 eV [26], and the work function of MAPbBr_3_ crystal is about 4.9 eV [27,28]. Once the perovskite and graphene are in contact, electrons in perovskite will flow to the graphene side, leaving the fixed positive ions to form a depleted space charge area. Thus, the energy bands of perovskite bend upward and create a Schottky barrier. This is well consistent with the rectifying *I*–*V* curve of the device. The concentration gradient drives electrons diffusing to the graphene side, while the built-in electric field generated by the space charge drives electrons to the perovskite side. Under illumination, the light will be mainly absorbed by the perovskite, and a number of electron–hole pairs are generated. These photo-induced carriers are quickly separated by the built-in potential, and give rise to the photocurrent.

Photoresponsivity (*R*) is one of the key parameters to evaluate a photodetection device, and can be defined as

(1) $$R=\frac{I_{ph}}{A\cdot P}=\frac{I_{light}-I_{d}}{A\cdot P}$$

where *P* is the irradiance power of incident light, *A* is the effective area of device (0.36 cm^2^ for our device, as shown in Section 2), and *I_light_* and *I_d_* are the photocurrent and dark current of the photodetector, respectively. Figure 4b,c show the responsivity of this perovskite/graphene heterostructure photodetector. When applying negative bias, the responsivity increases as the applied voltage increases, and the growth rate steps down under larger bias. This is mainly owing to the dependence of charge collection efficiency to bias voltage. When the bias voltage increases, the carrier collection efficiency increases, resulting in a rise of photocurrent. But if the bias voltage further increases, the collection efficiency of photo-induced carriers will approach its limit, resulting in the saturation of photoresponsivity [29]. The responsivity is no longer dependent on applied bias voltage. Almost all of the carriers generated by the incident light are sufficiently collected at the electrodes, and so the responsivity is only dependent on the light power density. As shown in Figure 4c, the responsivity increases as the light density decreases. This trend happened because a reduced light lowers the carrier concentration and the electron–hole recombination rate in the active layer [11]. The device reaches a highest responsivity value among our tests of ~0.9 mA/W at zero bias and ~20.2 mA/W at −10 V bias when the light density is 77.1 μW/cm^2^. This photoresponsivity is significantly superior to graphene-only photodetectors, which exhibit a photoresponsivity of 0.5 mA/W at a high bias voltage of 80 V [10]. When applying positive bias, the responsivity is much larger and exceeds a value of 0.73 A/W at +10 V bias and the same light density. However, the response time at positive bias is much slower than that at negative bias, which limits its applicability. 

The detectivity (D*) of the device can be calculated by using the following equation:

(2) $$D^{*}\approx R\sqrt{\frac{A}{2qI_{d}}}$$

where *R* is the photoresponsivity, *q* is the elementary charge, *I_d_* is the dark current, and *A* is the effective area of the device. As shown in Figure 4d, the detectivity of the photodetector is about 10^9^~10^11^ Jones among our tests and decreases as the light power increases. A value of about 4 × 10^10^ Jones can be obtained at zero bias for the light density of 77.1 μW/cm^2^, and the detectivity always exceeds 10^10^ Jones under the illumination power density of 77.1 μW/cm^2^, which is comparable with existing works [9,30].

**Figure 5:**
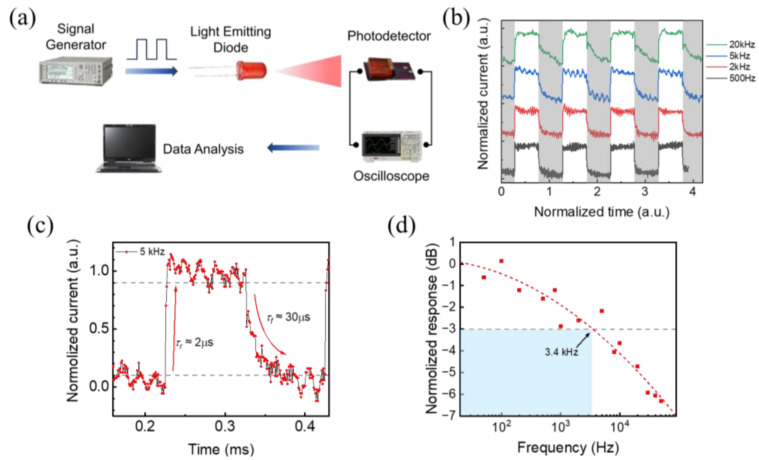
(**a**) Schematic of experimental setup for photo-switching behavior test. (**b**) Photoresponse of the device under alternating illumination of different frequency. (**c**) The transient response at 5 kHz alternating illumination. (**d**) Relation between the normalized responsivity and the optical modulation frequency.

The photo-switching behaviors of the photodetector are further studied by the experiment apparatus illustrated in Figure 5a. A signal generator arises periodic square wave signal, controlling a 405 nm light emitting diode (LED) to produce alternating light source. The photoresponse of the device is recorded by an oscilloscope. As shown in Figure 5b, the device exhibits repeatable and stable photoresponse under the high-frequency alternating illumination. The rise time (defined as the time for the signal to rise from 10% to 90% when light starts to shine) and decay time (defined as the time for the signal to fall from 90% to 10% when turning off the light) are measured under 5 kHz (Figure 5c), and the results are 2 μs and 30 μs, respectively, as shown in Figure 5c. For comparison, we also prepared a photodetector based on MAPbBr_3_ without graphene, which exhibits a response time in millisecond scale (Figure S3 in Supplementary Material). It is clear that the heterostructure device has a faster response time, which is mainly caused by the following: (1) the built-in electric field of the perovskite/graphene heterostructure facilitates the separation of electron–hole pairs and the transfer of charges; (2) the high mobility of graphene shortens the drift time of carriers to reach the electrode. In addition, the response time of our photodetector is shorter than other existing similar works, as shown in Table 1, which is mainly due to the good interface quality of the heterostructure brought about by the optimized wet transfer method. Figure 5d shows the normalized photoresponse under different-frequency modulated light. The 3 dB bandwidth of 3.4 kHz can be obtained from the curve.

We compared our perovskite/graphene photodetector with previous reported similar works, as shown in Table 1. Photodetectors with a field-effect-transistor (FET) structure, i.e., both of the two test electrodes are directly contacted to perovskites, exhibit high responsivities of several A/W, but their response time is over milliseconds and they cannot operate without external voltage bias. Photodetectors based on hybrid perovskite/graphene (graphene quantum dots mixed perovskite films, for example) material are a bit far from the topic we focused on. In these works, graphene-like elements are deeply mixed in perovskites as an additive rather than a separated layer. The remaining works in the table are more analogous with our work, of which the key part is also the perovskite/graphene heterostructure. Most of these photodetectors are self-driven and exhibit an ultrahigh responsivity over thousands of A/W, by virtue of the photogating effect. However, the response times of these photodetectors are relatively large. It can be seen that the self-driven heterojunction photodetector we prepared has an ultra-short response time. This performance should mainly be attributed to the optimized wet transfer method which results in a high-quality heterojunction interface, since the response time primarily depends on the recombination rate at the interface. The photoresponsivity of our detector is better than that of the graphene-only photodetector but is relatively low compared with other existing perovskite/graphene heterojunction photodetectors, as shown in Table 1. This can be mainly attributed to the low amounts of defects in graphene and the absence of photogating effect. Photo-induced holes are quickly recombined at the interface before the electrons reach the electrode, bringing about a low gain value.

## 4. Conclusions

In summary, we proposed an optimized wet transfer method, and constructed a centimeter-sized high-quality MAPbBr_3_ single crystal/graphene heterojunction photodetector by this method. Polydimethylsiloxane (PDMS) and saturated perovskite solution were used as supporting layer and transfer solvent. The heterojunction exhibits an archetypal rectifying behavior of Schottky contact. A responsivity of 0.9 mA/W with a detectivity over 10^10^ Jones was achieved at zero voltage bias under illumination of 520 nm wavelength. In addition, the detector shows an ultra-short response time of 2/30 μs by virtue of the good interface quality of the heterojunction. This study provides a facile method for fabricating a large-area perovskite/2D material hybrid device, and proves that MAPbBr_3_/graphene heterojunction could be a hopeful alternative for quick-respond light detection.

## Figures and Tables

**Table 1:** Performance comparison of photodetectors based on perovskite and graphene-like material reported in recent years.

Device Structure	Self-Driven	Photoresponsivity	Response Time	Ref.
MAPbI_3_ thin film/graphene	Yes	180 A/W @0.1 V 520 nm	87/540 ms	[9]
graphene	No	0.5 mA/W @80 V 1550 nm	-	[10]
MAPbI_3_ nanowire/graphene	Yes	2.6 × 10^6^ A/W @0.01 V 633 nm	≈5 s	[31]
MAPbBr_3_ single crystal (1.8 μm thickness)/graphene	No	1017.1 A/W @3 V 532 nm	50.9/26 ms	[32]
MAPbI_3_ thin film/graphene/TiO_2_/FTO	Yes	0.375 A/W @0 V 530 nm	≈5 ms	[33]
MAPbBr_2_I nano islands/graphene FET	No	6 × 10^5^ A/W @3 V 405 nm	0.12/0.75 ms	[34]
CsPbBr_3_ nanocrystal/graphene FET	No	3.4 A/W @4 V 405 nm	7.9/125 ms	[35]
CsPbBr_3_ QD (quantum dot)/graphene FET	No	27.5 A/W @1 V 405 nm	0.95/2.05 s	[36]
FAPbI_3_ QD/graphene FET	No	8.03 A/W @1 V 365 nm	0.68/6.79 s	[37]
MAPbI_3_/graphene QD	No	12 A/W @3 V 405 nm	0.17/0.19 s	[7]
CsPbBr_3_/N-doped graphene QD	No	3.21 A/W @3 V 520 nm	0.6 ms	[38]
MAPbI_3_/rGO (reduced graphene oxide)	Yes	721 mA/W @0.1 V 1064 nm	460 μs	[30]
MAPbI_3_/rGO/graphene/InGaAs/InP	Yes	340 A/W @1 V 500 nm	≈10 μs	[39]
MAPbBr_3_ single crystal/graphene	Yes	0.9 mA/V @0 V 520 nm	2/30 μs	This work

## Data Availability

Data are contained within the article and Supplementary Materials.

## Supplementary Materials

The following are available online at https://www.mdpi.com/article/10.3390/ma17112599/s1, Figure S1: (a) Schematic diagram of Au/MAPbBr_3_/Au photodetector. (b) Optical photograph of Au/MAPbBr_3_/Au photodetector; Figure S2: Current–voltage (*I*–*V*) characteristics of Au/MAPbBr_3_/Au photodetector; Figure S3: Response time of Au/MAPbBr_3_/Au photodetector at −1 V bias under illumination with light intensity of 857 μW/cm^2^ and wavelength of 520 nm.
