# Supplementary material for: Single Crystal Perovskite/Graphene Self-Driven Photodetector with Fast Response Speed
Source: Materials (Basel). 2024 May 28;17(11):2599. doi: 10.3390/ma17112599 (PMC11173920; doi:10.3390/ma17112599)
Supplement: Supplementary file 1 [file materials-17-02599-s001.zip › materials-2998944-supplementary.pdf]

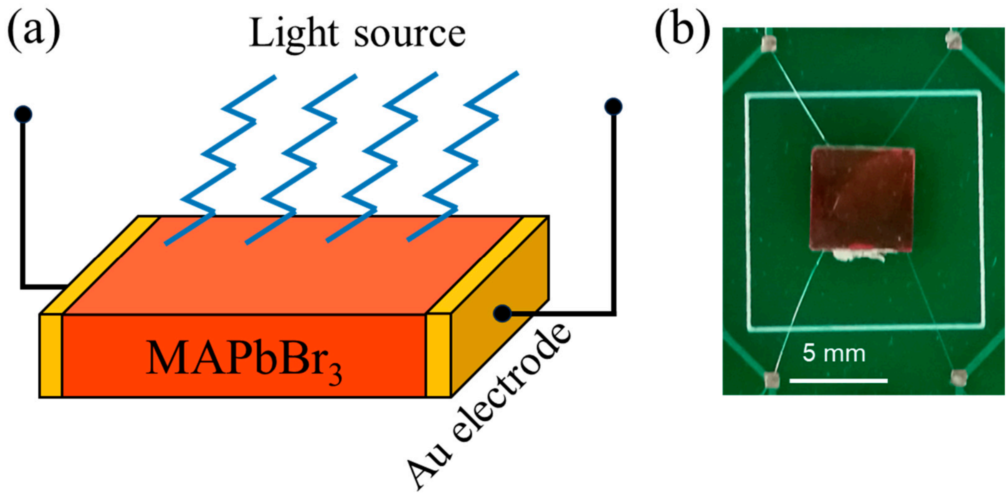

Figure S1. (a) Schematic diagram of Au/$MAPbBr_3$/Au photodetector. (b) Optical photograph of Au/$MAPbBr_3$/Au photodetector.

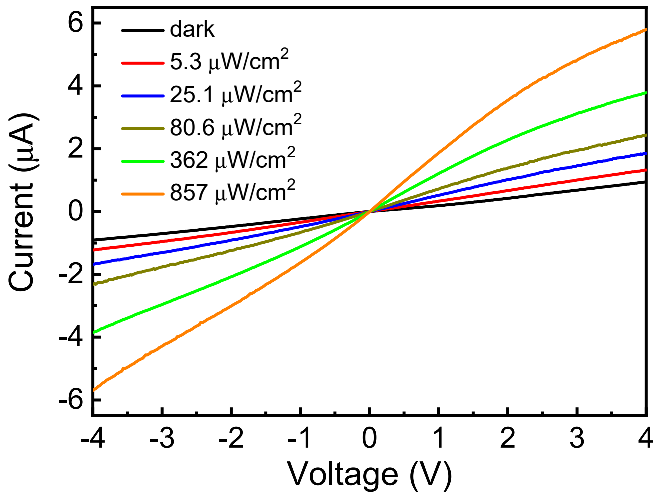

Figure S2. Current-voltage (*I-V*) characteristics of Au/$MAPbBr_3$/Au photodetector.

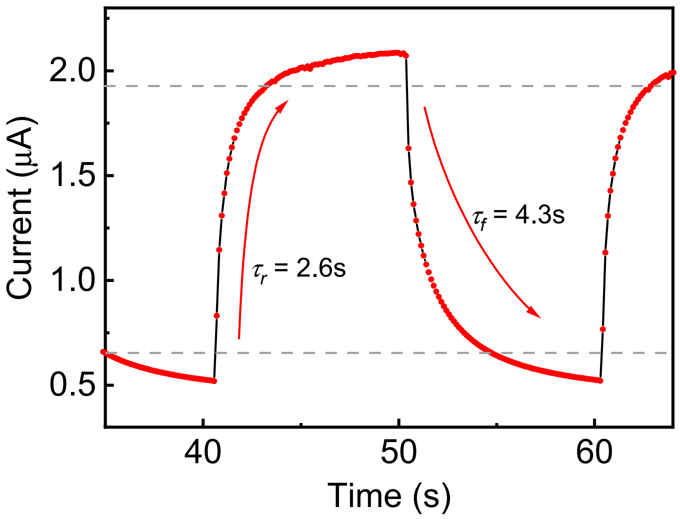

Figure S3. Response time of Au/$MAPbBr_3$/Au photodetector at -1 V bias under illumination with light intensity of 857 μW/cm^2 and wavelength of 520 nm.
